# Practical Management of Infants

**Published:** 1898-01

**Authors:** M. J. Crouch

**Affiliations:** Union, Ky.


### Practical Management of Infants.

BY M. J. CROUCH, M.D., UNION, KY.

Paper read before the North Kentucky Medical Society, October 11, 1897.

From a biological standpoint infancy and childhood are not only interesting periods of human existence, but of vast importance. These are periods of great activity. The structure and functions of the body undergo wonderful evolution. In considering this evolution, this wonderful development of a lower organism into a higher—for the infant as a vital unit is less differentiated than the child, the child less than the adult—we are not, or at least should not be, surprised at the immense amount of disease or the frightful mortality. Then, as clinicians and pathologists, we should be none the less interested. After all, what is disease but a departure from health? "The physiological processes differ from the pathological only in form and course." The one gives us our normal health and strength, the other disease and death. We are told that during these two periods there is more sickness and death than during all the other periods of life combined. For these and other reasons infancy and childhood will ever be of special interest to the physician and vital statistician. To-day I invite your attention to a general consideration of the first of these periods.

Infancy begins at birth and extends to the completion of first dentition. I shall merely mention some statistics to illustrate the mortality of the first period of life. These show that there are fifteen deaths in every one hundred infants before the close of the first year of life—four or five in every hundred during first month. Some give a larger death-rate, as one out of every ten, before close of first month. Statistics in England, on the continent of Europe and in this country vary. The mortality in the city and country vary greatly, but all unite in proving the terrible mortality of the first few years of human existence.

I think it may be safely stated that one-fourth of the children born in this country die before reaching the fifth year. The causes of this great mortality, and the means of preventing it, deserve careful consideration. We should give this subject more attention, at least more than we have. To judge by the mortality of early life, we physicians are all converts to the theory of Malthus, and are giving our talents to prevent the over-population of the earth, or else we are fighting a losing fight against nature and her invincible laws of "survival of the fittest." Who can say in regard to these things? I believe it is largely the latter, for the fact that disease exists at all is evidence that the organism is not in perfect harmony with its environment; that it is fatal is proof of the edict that it was unfit to survive.

I wish to explain here that the word "practical" is not used in this paper as the opposite of theoretical. My idea of this subject—or any other, for that matter—is that the best results are obtained by a thorough investigation and minute consideration of so-called "little things." If you are ignorant of this subject you may call these things theoretical if convenient. I trust you may find them practical. So what to the physician is practical management of the infant may and does frequently seem theoretical to those who have it in charge. Herein lies the difficulty, and in consideration of the cause of the high mortality of the infant, as well as of childhood, we have this ignorance as well as indifference to contend with, and they become right potent factors, too.

Other causes which go to make up this mortality are heredity, vices of formation, the infectious diseases, anti-hygienic conditions and teething.

The old saying that "an ounce of prevention is worth a pound of cure" is literally true in the treatment of diseases of children, and even more than true, for many diseases that can be prevented can not be cured.

Thus two potent causes of infantile mortality may not be cured, but they may and should largely be prevented. We refer to heredity and vices of formation. They must be considered together, for they are too closely related to properly be separated. To accomplish this it may be necessary to go forward a hundred years or more. But why not? Is it too theoretical? No! It is as practical as breathing if we but take hold of it, and it is worth the life effort of the greatest and best among us. The human mind can not have a more noble or grand conception than to apply the light and knowledge of the age to develop a better formed, more vigorous, and consequently a more beautiful and healthy race.

Infants whose parents are subjects of syphilis, tuberculosis and struma have little vitality and a large percentage succumb to their surroundings, if not directly to their diseases. These infants require all the more careful attention. They are victims of the present condition of things. I was about to say of civilization, but civilization may easily be cleared of this crime, as of many others. These things exist in our day by tolerance, not by necessity. Knowing of nature's laws we should assist, not thwart, her. She perfects her work by allowing only the "fittest" to survive. We should imitate by not allowing the subjects of tuberculosis, syphilis, inebriates, confirmed criminals and other degenerates to marry, or at least to procreate their kind. They have no moral right to do this crime against the human race; they should have no legal right to do so. I conceive it to be the duty of every one to help bring this about. When these things exist it is the duty of the true physician to do the best for these unfortunate ones that he possibly can. Congenital syphilis should be treated at once, and if the infant has escaped infection, it should be carefully guarded against.

If the syphilitic must marry and must have children, he should at least undergo a few years of proper treatment, as well as the mother, if she has already become infected, before bringing into the world poor, helpless, degenerate creatures.

Many of the internal malformations necessarily cause death. Some of these are no doubt due to syphilitic inflammation *in utero*. Ante-conception treatment would prevent this. Frequent abortions among the syphilitic is nature's verdict against the fitness of these infants to live, and no doubt saves us a still greater infantile mortality, a larger number of vices of formation, as well as lightens the burden of our eleemosynary institutions.

That vices of formation are due to maternal impressions is no longer tenable. The old women believe it, but there is no reason for enlightened physicians to believe such folk-lore. It is the duty of the family physician to disabuse the public of such notions. It is hard to unteach what we have taught. It is harder still to unlearn what we have learned. I was taught this, and ten years ago I used to warn my *enciente* patients against going where there would be a likelihood of meeting such objects as would mark or mar their fetus. In this case, as in all others, where we observe a fact that we do not understand, and consequently can not explain, we hide the fact in mysterious, unmeaning words and occult gestures. We used to tell our patients if they wanted well, beautiful and bright children to look at such children frequently, and keep beautiful, chubby, rosy-cheeked Cupid's pictures hung about their bed-chamber, etc. We know better now, and we tell these women, if they desire more beautiful, healthy, better developed children, they must begin with several generations of their ancestors, and marry only healthy, vigorous, well-formed, handsome men.

You say this is certainly theoretical, but I tell you it is strictly practical. Why? Create a healthy public sentiment this way, and you will see that people who are intelligent are quick to profit by any useful information. Those who are not intelligent, and degenerates who don't care for themselves, their children or remote posterity, may be helped in spite of themselves by proper legislation. Allow no one to marry without a clean bill of health, and you will soon bring about a better condition of things, and remove a distressing as well as potent cause of infantile mortality.

The good this would do is of inestimable value, not alone for the happiness and well-being of this generation, but the future existence, development, and good of the human family.

In regard to the death-rate from the infectious diseases, a great deal has been said. The table remains about the same, notwithstanding the claim of serum therapy. At best there is a lurking suspicion that, like "Pasteur Institutes," it increases the number of cases of certain diseases in direct ratio with the distance from the "institutes" using these serums, without any real decrease of mortality.

This running after fads, this dogmatism, this jumping to conclusions across unknown chasms and foolishly trying to build bridges with statistically-culled rubbish is certain to lay the practice of medicine open to the criticism that it represents no science. Let us take warning and "make haste slowly." Let us "prove all things and hold fast to that which is good." Applying these aphorisms to the subject of the germ theory, serum therapy and immunity, we will neither accept nor reject, but will conservatively investigate and use, however doubting, where time shall place them as necessary stones in the temple of science.

Prevention by isolation is certain. We know that intelligent isolation will protect our little patients. I want to emphasize this, for few people know what intelligent isolation means. It not only means to keep the child in a home that is not infected, but to keep things and persons infected out of that home. A great deal of ready-made clothing comes into the homes of the poor; some of these come from the city "sweat-shops," the hotbeds of infectious diseases. Proper legislation should compel the thorough disinfection of all ready-made clothing, as well as all public conveyances. Intelligent isolation does not mean that the father shall come into that home from public conveyances and other sources of contamination and rush to the nursery without change of clothing, or even bathing of hands, etc. Pseudo-protection is worthless. It is folly, and even criminal, to avoid, for instance, tuberculous milk and butter and make no effort to protect the helpless infant from the infectious kiss of the syphilitic and tuberculous. Tuberculosis may not be infectious, but the subject of advanced tuberculosis is no more a fit person to kiss an infant than an old glandered horse is a desirable companion for a healthy young colt. Promiscuous kissing is a dangerous practice at best. Only the purest, cleanest lips, fragrant with the breath of health, should dare to touch the lips of purity and innocence itself.

Then there is the prevalent idea that children must have certain contagious diseases because "they go easy with young children." I have known parties with this idea to take their helpless charges where they would be exposed to these diseases. They, no doubt, meant well, but how ignorant and wrong! Children thus exposed contract and often die of these diseases, to say nothing of other serious considerations, for there is no disease that an infant may contract that can be an advantage to it, and every one is a decided danger and unnecessary. Parents must also be taught that asafetida is a useless "hoodoo-charm," and *intelligent isolation the only preventive we have at the present time for the infectious and contagious diseases.*

Thus far we have considered some phases of the infant's formation, its hereditary tendencies, and the prevention of certain dangerous diseases. We believe that by the careful and extended application of these suggestions infantile mortality will not only be greatly lessened, but a more vigorous childhood insured.

In order that the disabled and weak infant be made stronger, the vigorous and sound retain its health and strength, that the complex structure and function of the developing infant be properly preserved and cultivated, we must continue to see that the child is properly fed, clothed and cared for. The best food for the infant is its mother's milk, provided that mother is healthy and the milk normal. The milk may be all right and the child healthy to begin with, yet it does not thrive; it is fretful, cries and tears about a great deal, does not sleep much. The mother, nurse, grandmother or somebody declares it is colicky, and must, of course, have soothing syrup—Castoria—and may be a hundred others not only worthless, but harmful, preparations. We must not take it for granted that the infant is colicky just because the women say it is. Not every child that frets and cries by the hour, by note or without note, by day or by night, has the colic. We may be sure of one thing, a child that is fretful and refuses to be nursed or quieted at all is not well; something is wrong. What that something is the physician must ascertain. If he can exclude the various hereditary and acquired infantile diseases, and concludes that the infant is the subject of acute indigestion or colic, his task is comparatively an easy one. The milk must be investigated first. To do this we note its specific gravity and reaction. We must see if there is much colostrum. If this is all as it should be we must look to the mother to see if she has any conditions that might injuriously affect her milk. Has she any unnatural discharge from the uterus, subinvolution, albumen in the urine, or other injurious conditions? Is she menstruating, pregnant, etc.? Is the husband subjecting the mother to undue sexual excitement? Has she indigestion or nervous trouble? In fact, every possible condition of the mother should be investigated and corrected before going to the infant for the cause of this indigestion. If the mother is all right the milk will be all right, as a rule, and the infant must, of necessity, be at fault, or our management erroneous. If the mother is in the habit of keeping the child almost constantly at the breast she must desist at once, no matter what the "old women" say. The infant does not require food oftener than every two hours during the day and once or twice at night until after the first month. At this time some will not require so frequent nursing. After the third month every three hours is sufficient for most infants. This will do, as a rule, but mathematical exactness is not necessary or required. Every case must be studied, as no two may be alike. If proper care along these lines does not prevent this trouble we must assist the feeble digestion of the infant. The use of properly-prepared enemas meet this requirement. Very little medicine is needed. Castor oil is the best for removing irritating contents from the bowels. Calomel should seldom if ever be used, and then only for a specific effect, not as a simple laxative or cathartic.

Special attention should be given to the diet of the nursing mother. One chemical and microscopical examination of the milk may not, as previously suggested, reveal important changes.

Certain foods eaten by the mother will colic the baby. So will certain drugs appear in the milk. This is a gentle hint from nature that we may use to the infant's advantage. In young infants it is seldom necessary to give a laxative, a good dose of castor oil, a little senna or salines for the mother being sufficient. So the mother should eat food especially rich in mineral matter needed for bone growth and fats for the nervous system, for the osseous and nervous systems are more rapidly developed during the period of infancy than the muscular. We should not lose sight of this important fact when, at about the sixth month, we begin to give a little artificial food. Of course, if the mother's milk is ample and no special reason exists, we should not be in a hurry to begin feeding. As soon, however, as the baby gets a few teeth it is well to gradually accustom it to other food than its mother's milk.

Weaning in all cases, if possible, should be a gradual process. It is a cruel mistake to suddenly deprive a child of the only food it has ever known. This is often done with the best of intention, and frequently results in harm to the child. Especially is this true in the cities during the heated term. Under these conditions no child should be weaned if possible to delay doing so. When we do begin to feed the baby, pure, fresh, healthy cow's milk should be the basis of its diet. Well-cooked light bread and plenty of good butter may be added; later on a little fruit jelly is also desirable. Then some ripe fruit like the banana and apple. Well-cooked cracked wheat, served with plenty of cream, should form a very important addition to the child's diet. It is almost unnecessary to suggest that the child should not be allowed to nurse and eat almost continually all day long, for any one should know better. However, this is the common practice as far as my experience goes. This should not be allowed. The child should nurse and have what additional food is allowed at regular intervals. Mashed potatoes and candy are not suitable food for infants—or even older children, as for that—unless in very limited quantities. *No prepared food for infants now on the market is equal to fresh, pure, healthy cow's milk.* If fresh cow's milk can not be had, properly prepared condensed milk will make a good substitute.

Feeding the infant is a most important part of its management. It is altogether too important to be left to the ignorant nurse or the equally uninformed parent if we expect to lessen infantile mortality.

Dressing the infant from the first should be under the immediate direction of the family physician. It seems to me that our way of dressing the new-born infant is wrong. Why not wrap it in a "swaddling-cloth" as of old, in the form of a good, warm flannel gown? Our present way is not only injurious, but cruel.

As soon as the child is born it should have its eyes carefully cleansed, umbilicus dressed and be wrapped carefully and snugly in the good warm folds of flannel. This is imperative in the case of weak infants. Reaction is feeble enough in the vigorous new-born infant, but in these weak puny ones the sudden exit from a sealed cavity of 100°F. to the ordinary temperature of the room is shock enough without any unnecessary exposure. The new-born infant is naturally protected by a sebaceous covering, which should not be removed for twenty-four hours. The child should be wiped free of blood and all other unusual collection of matter, but not scrubbed with soap and water and exposed until it is blue and shaking from the cold. I am surprised that this harmful practice continues. After the navel has healed a general bath with the temperature about blood-heat (100°F.) should be given daily, but continual due care taken to avoid exposure. The infant should be well protected against changes of temperature, but not by keeping it in a hot foul room, filled with fumes of tobacco and other noxious gases. A thousand times, no! Is it a wonder that the crowded unsanitary homes of the poor give us the largest infantile mortality, to say nothing of childhood diseases, finally sending out into the world poor, weak creatures, unfitted to cope with the world, easy victims of disease, warped and woven by those conditions of life, to become victims or criminals of our social order?

The infant should be dressed in flannel, plenty of it, heavy in winter and light in summer. The nursery should be the best lighted and ventilated room in the house. Every intelligent physician knows how important pure air and sunlight are to the public in general. We condemn the criminal ventilation of our public schools, churches and halls, as well as the private homes of the masses. It is bad enough to have to go into these miserably ventilated places at all, or have to remain only a few hours, and frequently this is only repeated at long intervals; but it is very different when one lives night and day in such an atmosphere. This is why children and women suffer more than men do, as a rule. Perhaps this is a potent reason that the female has not retained her vigor of nerve and muscle, as she has risen in the scale of civilization. To sleep in the close, foul air is bad enough when one breathes pure, refreshing atmosphere all day long; but infants live in this foul atmosphere. This condition is exaggerated in some homes, in the country as well as the city. This greatly debilitates the growing infant and assists much those terrible scourges of infancy—cholera infantum, entero-colitis, diphtheria and scarlatina.

Finally, before the infant enters childhood proper it must complete its first dentition. This period of teething is the great bugbear of the mother. If she has no sad experience of her own there are plenty around her who have. These old and young women can tell many a weird experience of teething. If a child is "teething," according to their experience, that will account for any and everything. If a child has "spasms" it is teething and its gums must be cut or lanced. If it has diarrhea "leave it alone, it is good for it, it is teething," and so on. This popular idea requires our attention, for it is erroneous and leads to much harm and many deaths. Doctors are to blame for this popular idea as well as many others. They used to lance the gums and treat "teething," too; but now that we know better we must teach the people better, and thus save a large number of human lives. Teething, we know, is a physiological process, and needs to be let alone. It can do no harm, nor does it cause any. If the child has eclampsia, look elsewhere for the cause; if it has diarrhea, treat the gastro-intestinal tract and regulate its diet. Hardly a summer passes that I am not called to see children dying from diarrhea, the so-called "summer-complaint," due to improper feeding. When I ask the parents how long the child has been sick they will admit a diarrhea all summer but will quickly add: "That was good for it, it was teething." That was why they did not consult a physician until too late.

So heredity, foul atmosphere, exposure to cold, heat, improper feeding, "teething," etc., not only make infantile mortality largely what it is, but these continue to operate through childhood and youth. Nor can their influence be said to stop there. The old adage, "As the twig is bent so is the tree," will apply here with all the force of truth and emphasis.

DISCUSSION.

Dr. Bagby: I want to compliment the essayist on the paper he has presented us. It is certainly original as well as practical. I want to say that I like the way he has divided his subject. It is well to place the period of infancy to completion of first dentition. Then the child is no longer an infant, at least as far as feeding is concerned.

The mortality of infancy has greatly improved in the last ten years, as well as general mortality. Improved management of the infant has done this. This is fortunate, for otherwise the decreased birth-rate would be alarming. People are better informed as well as more able to care for their children. I believe there is less syphilis, at least that is my experience. When I began practice, about thirty years ago, I frequently met cases. Now I rarely see one. I believe it will finally die out, like small-pox.

While we all have the same principles to guide us in the management of infants, we may have individual ideas about putting these into practice. Now I would not purposely expose an infant to any disease, but I think it would be better to have measles, for instance, while young, than later, as most people do have this common disease some time in their lives if not when young. Then it is a trivial affair; later, it is much more serious; and deprives one of work, or a woman may be pregnant, etc. Most other diseases we had better avoid.

I fully approve of the opinion that the mother's breast furnishes the best food for the infant, and cow's milk next. Cow's milk, of course, should be carefully prepared so as to resemble as near as possible woman's milk. I also like some of the prepared foods for infants. I use calomel and chalk for infants, rather think its use prevents jaundice.

Dr. Lassing: It seems to me that Dr. Crouch has about covered the ground as far as medical management is concerned. The suggestion of breeding a better race seems impracticable. It is out of range of society to control marriages. People will marry whom they please, and it will make no difference what we say.

About the disappearance of syphilitic diseases, I am sure I see less of it than I used to. I am not sorry, either, for it is a loathsome disease, and has done much to degenerate mankind. I don't know to what this decrease is due, whether increased virtue or diminished liability. It may be due to universal inoculation.

Dr. J. A. Loomis: I wish to criticise some parts of Dr. Crouch's excellent paper. In the first place, I think he places the mortality-rate too high. He must have taken them from hospital practice. I am sure they are too high for our country practice. I think the mortality-rate less than 5 percent. The mortality of infancy is certainly less than it used to be. I also think that antitoxine has a great deal to do with this; we certainly have a right to think so.

I think two hours too long an interval for an infant to do without food, especially weak ones. I would make it one hour and a half, and increase a half hour every six weeks. At one year is soon enough to begin feeding. The child will be stronger and have better digestion for deferring solid food this long.

My experience with syphilis has been very limited. I believe that the close examination of prostitutes has had a great deal to do with this. Whether we will be able to annihilate it is to be seen. Small-pox has become a thing of the past, and I hope we will ere long be able to say this about other terrible scourges.

I don't think children should have measles if we can help it—at least not before they have arrived at the age of eight or ten years.

I believe the idea of the essayist, in regard to prevention of tuberculous and syphilitic subjects from marrying, is good—very good. Family physicians could examine and give certificates. It is practicable and in course of time would be all right and good. It would then only be a matter of time when our whole race would be stronger. You physicians know of cases where you would not give certificates. Some cases you could refuse for time sufficient to give proper treatment at least. If we had a law to back us up it would be a great thing.

I am fully in accord with the suggestion to defer first ablution of infants. Perhaps it would be better to grease and wipe off. I also agree with Dr. Bagby in reference to calomel.

Dr. J. D. Violette: I think the paper covers the ground as completely as possible. I fully concur with the ideas advanced. We should, of course, be especially careful of infants whose parents have syphilis or tuberculosis. It is easier to tell the tuberculous child than the syphilitic. I have serious doubt if a child with a tuberculous mother should be nursed by its mother at all. Fresh air is certainly desirable. To this end, if no other, the baby carriage is a great invention.

I don't believe a child should have any disease if we can keep it from doing so. All disease is antagonistic to nature.

I wish to emphasize the suggestion in regard to the infant's first toilet. I don't think our infants should be rubbed, scrubbed and bundled up as in ordinary practice. A belly-band is necessary. Science has done much to help us care for infants.

Dr. Lassing. Does not the general longevity of man mean that infant mortality has been lessened? Is it not more due to less mortality in the beginning of life than adult prolongation?

Dr. Bagby: I see in life insurance statistics that adult life has been prolonged seven years.

Dr. J. D. Violette: I think all sanitary work has had something to do with this increased longevity. I believe there is a science as well as art in caring for infants. Every case is a law unto itself. We must use our judgment. I emphasize the early recognition of tuberculosis and syphilis in children.

Dr. B. K. Menefee: I have obtained some very valuable information. I think the idea advanced by the essayist in regard to the first bath of the infant is good. Dr. Jacobi, of New York, favors this. I believe the belly-band a relic of barbarism. I use it, but don't need it or approve of it. I think the proper dressing is borated cotton.

As to mortality-rate, I have had only three deaths in a hundred cases up to four and a half years of age. I believe a child should have no solid food until completion of first dentition; no starchy food until the child is old enough to digest to advantage this kind of food.

In nearly every case of troublesome diarrhea I hear the old women say it is due to teething and so neglect them. Improper feeding causes it, of course, and not teething. In regard to castor oil for infants, it is all right, but I prefer calomel and soda, and think it the remedy *par excellence* unless we have hard feces, then give glycerine suppository.

I don't believe in exposing the child to any disease. Children should be especially guarded from whooping-cough and scarlatina.

It is a good plan to put a little salt, sugar or saccharine, with some lime, in cow's milk, then properly dilute, and you have the best substitute for mother's milk. It is a good plan where we use cow's milk to use from several cows, so as to avoid trouble from heat and pregnancy. I call to mind a case where a child took severe diarrhea, and shortly died, from using a cow's milk that was in heat.

A child should not be rocked or jolted on the knee much. It is wrong. It should also be taken to the dentist to have its teeth looked after.

Dr. J. S. Adams (dentist): I should not pull first teeth; if decayed, fill them. There are good reasons for this, which you all understand better than I do, no doubt. People generally neglect children's teeth, which is wrong.

---

For thirty-two acres in New York City there are 986.4 people to the acre. The nearest approach to this congested condition is a little spot in Bombay, where there are 759.66 to the acre. The densest London district has only 365.3 people to the acre.

